# Clustering protein environments for function prediction: finding PROSITE motifs in 3D

**DOI:** 10.1186/1471-2105-8-S4-S10

**Published:** 2007-05-22

**Authors:** Sungroh Yoon, Jessica C Ebert, Eui-Young Chung, Giovanni De Micheli, Russ B Altman

**Affiliations:** 1 Computer Systems Laboratory, Stanford University, Stanford, CA 94305, USA; 2 Department of Genetics, Stanford University, Stanford, CA 94305, USA; 3 School of Electrical and Electronic Engineering, Yonsei University, Seoul 120-749, Republic of Korea; 4 Integrated Systems Center, Swiss Federal Institute of Technology (EPFL), Lausanne, CH-1015, Switzerland; 5 Intel Corporation, 2200 Mission College Blvd., Santa Clara, CA 95054, USA

## Abstract

**Background:**

Structural genomics initiatives are producing increasing numbers of three-dimensional (3D) structures for which there is little functional information. Structure-based annotation of molecular function is therefore becoming critical. We previously presented FEATURE, a method for describing microenvironments around functional sites in proteins. However, FEATURE uses supervised machine learning and so is limited to building models for sites of known importance and location. We hypothesized that there are a large number of sites in proteins that are associated with function that have not yet been recognized. Toward that end, we have developed a method for clustering protein microenvironments in order to evaluate the potential for discovering novel sites that have not been previously identified.

**Results:**

We have prototyped a computational method for rapid clustering of millions of microenvironments in order to discover residues whose surrounding environments are similar and which may therefore share a functional or structural role. We clustered nearly 2,000,000 environments from 9,600 protein chains and defined 4,550 clusters. As a preliminary validation, we asked whether known 3D environments associated with PROSITE motifs were "rediscovered". We found examples of clusters highly enriched for residues that share PROSITE sequence motifs.

**Conclusion:**

Our results demonstrate that we can cluster protein environments successfully using a simplified representation and K-means clustering algorithm. The rediscovery of known 3D motifs allows us to calibrate the size and intercluster distances that characterize useful clusters. This information will then allow us to find new clusters with similar characteristics that represent novel structural or functional sites.

## Background

With the successful sequencing of the human genome and the genomes of many model organisms, attention has focused on determining the function of the protein products derived from the genome. Function is difficult to define precisely and can be considered at many levels, from the molecular to the organismal or even the population level. General function can often be assigned based solely on sequence analysis and similarity (using homology arguments). However, these methods are imperfect, as functions of proteins with common descent may evolve away from one another [1]. The molecular functions associated with a three-dimensional (3D) protein structure are somewhat less diverse and arguably more manageable: they include the basic activities of binding, catalysis, structural support, structural dynamics, and other functions that can be defined with respect to particular arrangements of atoms, the physical forces and environment they create, and the dynamics that result. Thus, when structure is available, the discussion of function can often become more detailed.

Structure-based methods for predicting function have recently become important in the context of structural genomics initiatives. Traditionally, protein structures have been determined for biologically critical molecules where biologists have required knowledge of structure in order to understand the details of mechanism or interactions. These proteins were often studied extensively prior to structural investigations, and so there was significant functional information available. Upon solving the structure, there were also many assays available to test the protein (and its mutants) in order to further probe the function. With the rise of structural genomics initiatives, there is an increasing number of protein structures available for which there is little or no functional information [1]. Indeed, these proteins are often prioritized precisely because they represent relatively unexplored segments of the protein structural space. Thus, methods are needed that can look at a novel 3D structure and label areas of potential functional significance (e.g., active sites or binding sites).

Methods for structure-based function prediction are diverse. Many rely on global fold recognition in order to more sensitively detect family relationships [2], but we stress in this brief review those methods that focus on local segments of 3D structures. The FFF [3] and JESS [4] methods build 3D templates that specify amino acid residues and allowable geometric relationships to define sites of interest, using examples from which the models are constructed. The evolutionary trace (ET [5]) method and ConSurf [6] both take advantage of evolutionary information to highlight regions of 3D structure with high probability of functional importance. THEMATICS [7] is a program that uses electrostatic environment and associated statistics of theoretical microscopic titration curves to highlight regions of likely enzymatic activity. Query3d [8] is a system that stores annotation information for all residues in a protein and allows this information to be queried and compared in the context of looking for similar residue environments. Some methods have moved away from representations focused on amino acids and use representations that are both spatial and sensitive to chemical groups. For example, SuMo [9] uses stereochemical groups to represent protein environments. Similarly, we have reported on the FEATURE method [10-13], which represents a microenvironment as a set of concentric shells (of radius 6–10 Angstroms) around a central point. Physical and chemical properties within these shells (as summarized in Table 1) are counted in sites of interest, and their counts are compared with control nonsites. In this way, FEATURE develops a statistical model of the three-dimensional distribution of properties that are distinct for the sites of interest and are not defined based on sequence features. FEATURE has been used to characterize sites such as calcium binding [13], ATP binding [12], serine protease active sites [14], and others [15]. Up until now, FEATURE has relied on supervised machine learning to build models based on known examples of a site. In this paper, we investigate the possibility of using FEATURE's representation of sites to perform unsupervised machine learning (clustering) on all potential sites in a nonredundant subset of the Protein Data Bank (PDB, [16]). The ability to cluster sites would allow us to identify previously unrecognized similarities between 3D sections of diverse proteins and could provide important clues to the function of these sections. While previous studies have used unsupervised learning techniques to discover functional relationships among structural fragments extracted from proteins [17-19], our approach differs in that we examine the relationships among the local environments around residues.

We describe our results in performing a clustering of 3D protein environments, represented as FEATURE vectors, on a subset of the PDB with no two structures having more than 50% sequence similarity. FEATURE vectors have previously been used to gain insight into a local environment's potential function by finding similar environments in other protein structures [20,21], and we extend this approach by improving the distance measure used to compare vectors and by progressing from classification of individual sites of interest to a full clustering of a nonredundant subset of the PDB. In particular, the aims of the current studies include the following:

• To develop a reduced representation of protein sites that can be rapidly compared using a distance metric during clustering but which yields tight and separated clusters

• To test this reduced representation using a fast K-means clustering algorithm employing a novel distance metric and a biologically informed selection of initial cluster centers

• To validate the resulting clusters with respect to their quality and their ability to rediscover known "positive control" clusters

In particular, we find that our method is able to detect clusters that capture known 1D motifs from PROSITE [22]. These 1D motifs were not provided to the algorithm, and this result suggests that the reduced representation and cluster algorithm are finding clusters that are robust and biologically relevant. They also provide statistics that will allow us to analyze and prioritize novel clusters in future work.

## Results

**Figure 1:**
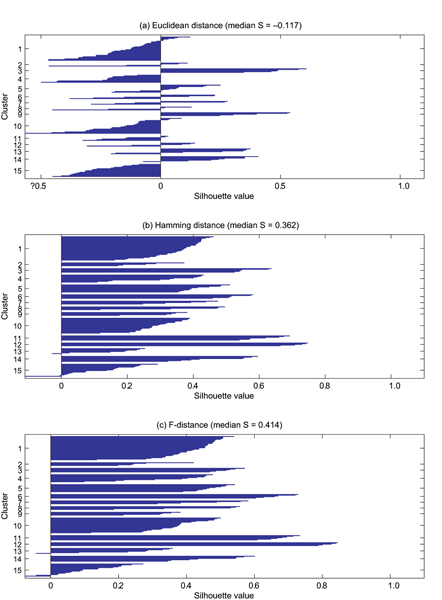
**Comparison of distance metrics**. We evaluated the performance of different distance metrics using the median silhouette value (S). A silhouette value represents the clustering quality for each object in a cluster as a continuous number between +1 (perfectly clustered) and -1 (the opposite). In order to evaluate the performance of various distance metrics, we calculated the silhouette values for each object in 15 training clusters based on previously validated FEATURE models. (a) The Euclidean distance gave a negative median silhouette value (-0.117), indicating that it is not suitable for clustering FEATURE vectors. The distance was calculated using FEATURE vectors in their original representations before any preprocessing occurred. (b) After converting the FEATURE vectors in the 15 clusters into their binary representations, we obtained better separation between clusters (median silhouette value of 0.362). (c) The weighted Hamming distance (called F-distance) produced an even better result (median silhouette value of 0.414) than the unweighted Hamming distance and was thus selected for clustering of the entire dataset of binary FEATURE vectors.

In order to ensure that protein environments with known similarities would cluster together using our FEATURE vector representation, we compared the results of clustering vectors from environments known to be similar (from previously built FEATURE models) using several different distance measures. We use the silhouette value (see Methods section) in order to quantify clustering quality for each object in a cluster by a continuous number between +1 (perfectly clustered) and -1 (the opposite). The plot of these values, termed the silhouette plot, shows tight and separate clusters as blue bars to the right, whereas loose or overlapping clusters appear as blue bars to the left (as described in the Methods section). Figures 1A and 1B compare clustering results using the Euclidean distance between the original FEATURE vectors to the Hamming distance between binary vectors. The Hamming distance between two binary vectors is defined as the number of coordinates in which the vectors' values differ. As seen in the plots, clustering vectors in their binary representation produces a better result in terms of the median silhouette values. In Figure 1C, we show that the F-distance (a weighted version of the Hamming distance) outperforms the Hamming distance and produces clusters with much better definition in terms of the silhouette value. On average, a cluster contains 1.06 residues from each protein chain represented in the cluster, indicating that the potential similarities between overlapping local environments do not dominate the clustering results.

For the full clustering, we used a single-processor 3.6-GHz P4 machine with 4 GB of memory running the Linux operating system. The total runtime of clustering nearly two million vectors was on the order of a few hours.

**Figure 2:**
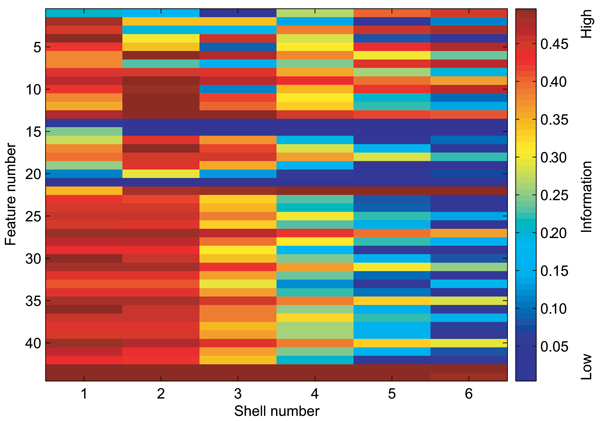
**Distribution of *f*_*i *_over all the FEATURE vectors used**. The weight used in the F-distance, *f*_*i*_, is related to the information content of feature *i*, and weights the distance computed between two FEATURE vectors. The values of *f*_*i *_are calculated from the distribution of ones and zeros in feature *i *over the entire two million FEATURE vectors used. This heat map shows the values of *f*_*i *_over all 44 features in each of the 6 shells.

Figure 2 shows a heat map of the values of *f*_*i*_, a measure of information content after normalization, over all the 44 features and the 6 shells used. The vast majority of properties have high information content at low to medium radii, and many properties have high information content even at 6 to 7.5 Angstroms.

### Initial validation of clusters

**Figure 3:**
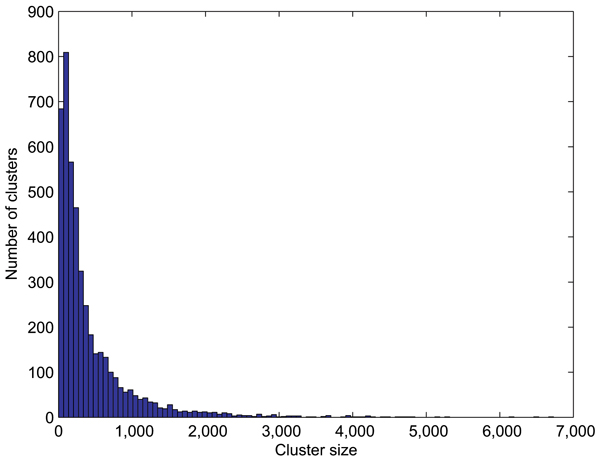
**Distribution of cluster sizes**. The number of residues in each cluster ranges from as few as 2 to as many as 6,731. The mean and median sizes are 437.2 and 232, respectively, and the standard deviation is 589.8. As discussed in the text, the long tail may represent internal hydrophobic environments.

We have defined a distance metric between two binary vectors based on the Hamming distance that takes the information content of each feature into account (see Methods section). We call this weighted Hamming distance the F-distance. In our clustering, the mean distance between clusters (intercluster distance) was 0.210 ± 0.197 F-distance units. The mean distance of vectors within clusters (intracluster distance) was 0.118 ± 0.028 F-distance units. Thus, as expected from the trial silhouette plots using the F-distance in Figure 1, the resulting clusters are generally tight and separated. Figure 3 shows a histogram of cluster size. The number of FEATURE vectors in each cluster ranges from as few as 2 to as many as 6,731. The mean and median sizes are 437.2 and 232, respectively, and the standard deviation is 589.8.

**Figure 4:**
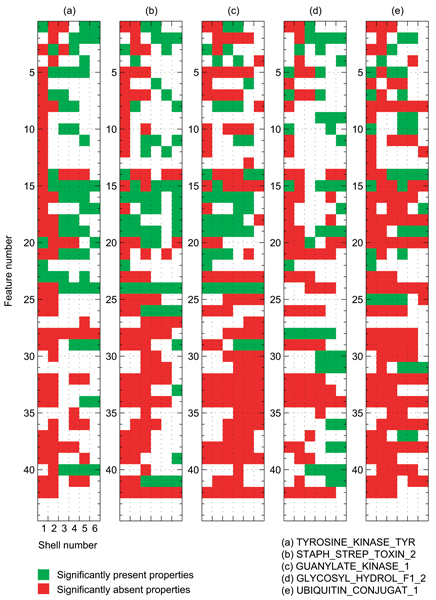
**Fingerprints of over- or underrepresented features**. This figure presents fingerprints of the features that are over- or underrepresented in each of the five validation clusters described in the text. The descriptions of the features are listed in Table 1. Green cells indicate the features that are significantly overrepresented with respect to the background of all two million feature vectors (P < 0.001). Red cells indicate features that are underrepresented. We used the two-sample test for binomial proportions for p-value computation. The rows and columns correspond to feature numbers (see Table 1) and the shell numbers, respectively.

In terms of biological validation, Figure 4 presents fingerprints of the features listed in Table 1 that are over- or underrepresented in each of the clusters described below with respect to the background of all two million feature vectors. Since some variation among environments sharing the same PROSITE annotation is expected, we do not anticipate that all examples of a given motif will cluster together. We present five examples in which at least 75% of the hits to a PROSITE pattern among the 9,600 protein chains used in this study occur in the same cluster. All these clusters have additional unannotated residues. These may represent novel predictions of shared function or they may be cases of related but different functions. Assessment of novelty will be addressed in future work. Our focus here is to evaluate the validity of the clustering approach.

### Tyrosine protein kinases specific active-site signature

**Figure 5:**
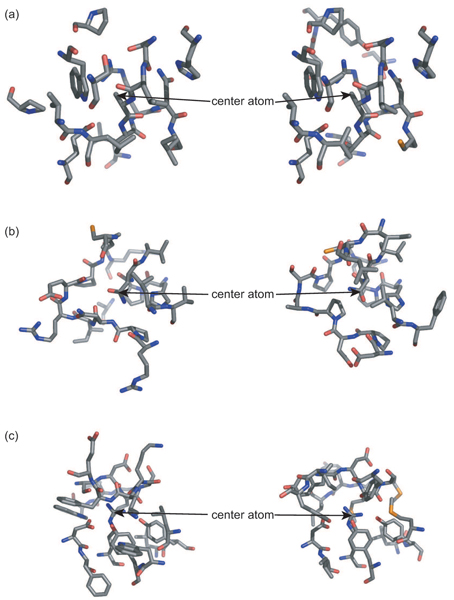
**Structural examples of environments from validation clusters**. Examples of pairs of environments from three of the validation clusters discussed in the text are shown. The structures were oriented by superimposing the PROSITE patterns, and the arrows indicate the atoms around which the microenvironments were centered. All residues containing atoms within the 7.5-Angstrom environment are depicted. The three comparisons show varying degrees of similarities among environments in the same cluster, ranging from nearly identical (a) to somewhat diverse (c). (a) The environments in the cluster containing residues from the PROTEIN_KINASE_TYR PROSITE motif are quite similar (top: PDB identifier 1fvr; bottom: 1luf). (b, c) The UBIQUITIN_CONJUGAT_1 (top: 1ayz; bottom: 1wzv) and STAPH_STREP_TOXIN_2 (top: 1aw7; bottom: 1ck1) clusters show greater degrees of structural variability. These images were produced using PyMol [28].

Thirteen of the 17 TYROSINE_KINASE_TYR PROSITE pattern (accession number PS00109) hits among the protein chains used in this study are contained within a single cluster. There are 346 total residues clustered together. Of the 15 residues in this cluster that have PROSITE annotations, only 4 do not belong to this motif. The average sequence identity among the proteins in this cluster with the tyrosine protein kinase motif is 31.4 ± 4.8%. Figure 4A shows a fingerprint of the features listed in Table 1 that are over- or underrepresented in this cluster with respect to the background of all two million feature vectors, and Figure 5A shows a comparison of the environments around two residues from the cluster that share the TYROSINE_KINASE_TYR annotation. All the residues in the cluster annotated with this PROSITE pattern are centered around alanines, and they share a great deal of structural similarity even though only one-half of the residues in the environment are contained within the PROSITE pattern itself.

### Staphyloccocal enterotoxin/streptococcal pyrogenic exotoxin signature 2

Ten examples of the STAPH_STREP_TOXIN_2 PROSITE motif (accession number PS00278) occur in our dataset, and nine of these occur in a single cluster (Figure 4B). There are 275 total residues in this cluster, 6 of which have PROSITE annotations other than STAPH_STREP_TOXIN_2. The average sequence identity among the proteins in this cluster with this pattern is 20.3 ± 8.4%. Three clusters are required to capture all 10 instances of this motif in our data set. The two examples of environments in this cluster around residues that participate in the STAPH_STREP_TOXIN_2 motif (Figure 5C) exhibit greater structural diversity than do the environments from the other validation clusters described here. Fewer than one-third of the residues in these two environments are located within the motif.

### Guanylate kinase-like signature

Four of the five hits to the GUANYLATE_KINASE_1 PROSITE motif (accession number PS00856) within our dataset are represented in a single cluster (Figure 4C). There are 162 total residues in this cluster, and 3 residues have differing PROSITE annotations. The average pairwise sequence identity among the proteins in this cluster with the guanylate kinase-like signature is 21.7 ± 6.0%.

### Glycosyl hydrolases family 1 active site

All the seven hits to the GLYCOSYL_HYDROL_F1_2 PROSITE pattern (accession number PS00572) in our dataset are represented in a single cluster (Figure 4D). Of the 151 residues in this cluster, 6 have PROSITE annotations other than GLYCOSYL_HYDROL_F1_2. The average pairwise sequence identity among the proteins with the glycosyl hydrolase family 1 active site motif is 31.6 ± 5.5%.

### Ubiquitin-conjugating enzymes active site

Eight of the 10 hits to the UBIQUITIN_CONJUGAT_1 PROSITE pattern (accession number PS00183) occur in the same cluster (Figure 4E). Of the 362 total residues in the cluster, 7 have alternate PROSITE annotations. The average pairwise sequence identity among the proteins with the ubiquitin-conjugating enzyme active site motif is 26.2 ± 4.2%. Figure 5B shows two examples of environments around asparagine residues contained in the UBIQUITIN_CONJUGAT_1 motif. Despite the fact that the cysteine residue toward the top of this figure is annotated in the PROSITE database as the catalytic residue, it is in the outskirts of the environment. Because active sites are often dynamic, regions that are slightly removed from the center of catalytic activity may show stronger conservation than the active site itself. Fewer than one-half of the residues in the environments are located within the PROSITE motif.

### Statistical significance of the validation clusters

Since it may have been the case that the five validation clusters described above could have occurred by chance, we repeatedly reassigned the two million feature vectors into our clusters randomly and assessed the segregation of residues with the same PROSITE annotation into clusters. As all of the PROSITE patterns associated with the validation clusters reported above have at least five hits in our dataset, we limited our analysis to PROSITE patterns with at least five occurrences. In approximately 13% of 50,000 trials, we observed one case where at least 75% of the hits to a PROSITE pattern occurred in a single cluster. The probabilities of obtaining two or three such clusters were 0.7% and 0.02%, respectively. A random trial in which four PROSITE patterns were each predominantly captured in a single cluster occurred only once, and we never observed five patterns to cluster according to these criteria. Thus, the results reported above (five examples of PROSITE patterns having at least five hits that are each predominantly captured in a single cluster) are statistically significant.

We also evaluated the overall performance of the clustering algorithm by determining how well each PROSITE pattern with at least three hits in our dataset clustered. For each pattern, we identified the cluster in which the highest percentage of hits is represented. On average, 67.0 ± 22.3% of the hits to a pattern are represented in the cluster that best captures that pattern. For the 50,000 random clusterings described above, this number drops to 38.5 ± 0.5%. If we exclude all patterns with fewer than five hits, 57.4 ± 21.2% of hits occur in the best cluster, whereas only 28.6 ± 0.5% are expected to cluster together by chance.

## Discussion

The major findings of this study are as follows. First, a binary representation of the FEATURE vectors with a weighted distance metric does not lose significant information necessary for clustering and in fact improves the compactness and separation of clusters in a manual assessment of 15 FEATURE models built by hand (as shown in Figure 1). This is a surprising finding since one might guess that continuous valued features would contain more information. However, the simple division of discrete variables into "zero" or "not zero" and continuous variables into "less than median" and "more than median" outperforms the continuous features. Further, the F-weighting scheme that we employed upweights features with good discrimination power when computing distances while retaining the binary representation, as shown in Figure 2. These results give us confidence that real (positive control) 3D environmental clusters are recognizable by our distance metric.

Second, we have shown that the computational complexity of K-means clustering on 2 million residues is feasible, with clustering completed within hours on a single modest CPU. The K-means algorithm is amenable to parallelization, and so we should be able to expand the granularity of our representation to not only include more atoms per residue, but also allow characterization of the "empty" space near residues, such as in pockets of proteins. The K-means algorithm reaches local minima, so we have begun to experiment with hierarchical K-means (e.g., recursive 2-means clustering) until the clusters are sufficiently small to allow hierarchical clustering. These approaches may be more robust. The choice to evaluate values of K up to 5,000 is a somewhat arbitrary one. We eventually chose to work with K = 4,550. The histogram in Figure 3 shows that the median cluster size is 232, but there is a very long tail. All our positive validation examples come from clusters in the range of 150 to 350 vectors, and so this may be a clue to focus our search for novelty in clusters within that range. A larger K might allow us to split some of the very large clusters. We can also experiment with different strategies for seeding the initial centers. It may be that our focus on surface points (which are likely to be involved in protein function) left few centers that match internal, hydrophobic environments. The large clusters may simply be undifferentiated hydrophobic environments, which account for a large volume of the total protein environments. A preliminary analysis of clusters with more than 1000 residues indicates that the top three amino acids occurring in those clusters are leucine, valine, and alanine.

Third, our method has found clusters that are enriched for PROSITE motifs, which are based primarily on sequence analysis. Of course, these motifs have 3D conformations in the associated protein structures, and we have shown previously that these 3D conformations can be used as a seed to create a FEATURE model that is more sensitive than the 1D sequence motif [23]. Not surprisingly, the addition of 3D information improves the motif because not only the identity of the amino acids, but also their relative positions can be encoded. In this work, we have shown further that a clustering of amino acid environments based on the FEATURE radial concentric shells encoded in a weighted binary vector can detect the similarity of the 3D environments associated with PROSITE motifs. Figure 4 shows the detailed FEATURE fingerprint associated with these four clusters. Each is unique and picks up a different PROSITE motif (as well as other positives that require further investigation). If these motifs had not been previously known based on sequence analysis, our clustering would now have suggested their existence. This forms the basis of our optimism that a complete analysis of the resulting clusters will yield not only other known 3D fingerprints (such as those already created for WebFEATURE [15] and by other methods), but also biologically novel motifs. In order to discover and characterize these novel motifs, we will need to systematically characterize the cluster properties of the clusters corresponding to known motifs and then use these properties to identify other promising leads. For example, in our five validated PROSITE clusters, only a small fraction of the vectors are annotated by PROSITE. Are these closer to one another than to the other cluster members, or are they scattered uniformly throughout the cluster? If they are closer to one another, then we may need more cluster centers to distinguish subgroups, or a hierarchical clustering to show the relationships between subclusters. If they are interspersed, it may suggest that we have found some unrecognized sites with environments that are very similar to those of the PROSITE-annotated residues. Thus in future work, we will need to dissect all the clusters in order to identify the key features of novel functional clusters.

## Conclusion

We have developed a reduced representation for the environment around an amino acid in a protein. The representation is binary and can be used in a weighted form to outperform ostensibly "higher information content" representations in identifying compact and separable clusters. A preliminary K-means clustering of a 50% nonredundant subset of the PDB produced 4,550 clusters, some of which clearly capture the key features of known PROSITE motifs as manifested in solved 3D structures. These results suggest that further refinement and analysis of these clusters may provide previously undetected functional sites and metrics for recognizing them.

## Methods

### Data preparation and preprocessing

We downloaded a list of approximately 9,600 nonredundant protein chains in the PDB [16,24]. No two structures in this data set share greater than 50% sequence similarity. From these structures, we derived 1,992,567 FEATURE vectors. Each vector represents the 44 physicochemical environments listed in Table 1 measured along 6 concentric shells with radii of 1.25 Angstroms, yielding a total of 244 dimensions in an environment with a radius of 7.5 Angstroms. Vectors for amino acids with aromatic rings are centered at the centroid of the rings, and vectors for hydrophobic residues are centered at the beta carbon. A hypothetical beta carbon is constructed for glycines based on idealized backbone geometry. For amino acids with polar side chains, the FEATURE vector is centered at the centroid of the functional group containing the polar atoms. For instance, the vectors for serines, threoines, and tyrosines are centered at the oxygen atom of the hydroxyl group. Since tyrosine contains both an aromatic group and a hydroxyl group, it is represented by two separate feature vectors. We also constructed two feature vectors for tryptophans; these are centered at the beta carbon and at the centroid of the aromatic rings.

As previously reported, FEATURE vectors are a combination of continuous and discrete variables. This makes the definition of a distance metric challenging. In order to simplify the vectors, we converted each FEATURE vector into a binary form. For discrete variables, the zero values were kept, and the non-zero values were replaced with 1. For continuous variables, the values less than the median value among all two million feature vectors were set to zero, and the others were set to one.

We adopted this preprocessing method because clustering results on 15 FEATURE models previously built manually [3,4] showed that the FEATURE vectors in the binary representation produced better clusters than the originals in terms of the silhouette value [25].

The silhouette value *s *is defined as:

s(i)=min⁡k{b(i,clusterk)}−a(i)max⁡{a(i),min⁡k{b(i,clusterk)}
 MathType@MTEF@5@5@+=feaafiart1ev1aaatCvAUfKttLearuWrP9MDH5MBPbIqV92AaeXatLxBI9gBaebbnrfifHhDYfgasaacH8akY=wiFfYdH8Gipec8Eeeu0xXdbba9frFj0=OqFfea0dXdd9vqai=hGuQ8kuc9pgc9s8qqaq=dirpe0xb9q8qiLsFr0=vr0=vr0dc8meaabaqaciaacaGaaeqabaqabeGadaaakeaacqWGZbWCcqGGOaakcqWGPbqAcqGGPaqkcqGH9aqpdaWcaaqaamaaxababaGagiyBa0MaeiyAaKMaeiOBa4galeaacqWGRbWAaeqaaOGaei4EaSNaemOyaiMaeiikaGIaemyAaKMaeiilaWIaem4yamMaemiBaWMaemyDauNaem4CamNaemiDaqNaemyzauMaemOCai3aaSbaaSqaaiabdUgaRbqabaGccqGGPaqkcqGG9bqFcqGHsislcqWGHbqycqGGOaakcqWGPbqAcqGGPaqkaeaacyGGTbqBcqGGHbqycqGG4baEcqGG7bWEcqWGHbqycqGGOaakcqWGPbqAcqGGPaqkcqGGSaaldaWfqaqaaiGbc2gaTjabcMgaPjabc6gaUbWcbaGaem4AaSgabeaakiabcUha7jabdkgaIjabcIcaOiabdMgaPjabcYcaSiabdogaJjabdYgaSjabdwha1jabdohaZjabdsha0jabdwgaLjabdkhaYnaaBaaaleaacqWGRbWAaeqaaOGaeiykaKIaeiyFa0haaaaa@750B@

where *a(i) *is the average distance from the *i*^th ^point to the other points in its cluster, and *b(i, cluster*_*k*_*) *is the average distance from the *i*^th ^point to points in another cluster *k*. A silhouette value is used in order to quantify clustering quality for each object in a cluster by a continuous number between +1 (perfectly clustered) and -1 (the opposite).

### Clustering FEATURE vectors

We used the K-means clustering algorithm to cluster the binary vectors. We first select K initial centers in a manner designed to bias the selection toward likely functional sites (see below). The distance metric (F-distance, as above) is then used to assign each vector to one of the centers. After all vectors are assigned, new cluster centers are computed as the average of all the assigned vectors. The average value for each dimension is determined by a voting method – if there are more ones, then the average is set to one, or else it is set to zero. The procedure is terminated when the cluster centers do not move more than some predefined cutoff value.

Since the K-means algorithm is an expectation-maximization (EM) algorithm that can find only local optima, it is sensitive to the location of initial centers [26]. In order to improve our selection of initial center locations, we generated and examined distributions for solvent exposure and atom density over all feature vectors. Based on these distributions, we defined four classes of points: (1) low atom density and high solvent exposure; (2) low atom density and low solvent exposure; (3) high atom density and high solvent exposure; and (4) high atom density and low solvent exposure. Since functional sites, such as surface pockets, are most likely to belong to the first class, 50% of our initial cluster centers were randomly selected from it. Ten percent of the cluster centers belong to the second class, 20% belong to the third class, and 20% belong to the fourth class. We varied the number of cluster centers (K) between 300 and 5,000 and found that K = 4,550 provided the best correlation with known functional sites (as discussed in the Results section).

We define a weighted Hamming distance, the F-distance, as a distance metric to be used in the binary vector space. The F-distance between two *n*-dimensional binary vectors *X *= (*X*_1_, *X*_2_,...,*X*_*n*_) and Y = (*Y*_1_, *Y*_2_,...*Y*_*n*_) is defined as follows:

F−distance=1n∑i2fi|Xi−Yi|
 MathType@MTEF@5@5@+=feaafiart1ev1aaatCvAUfKttLearuWrP9MDH5MBPbIqV92AaeXatLxBI9gBaebbnrfifHhDYfgasaacH8akY=wiFfYdH8Gipec8Eeeu0xXdbba9frFj0=OqFfea0dXdd9vqai=hGuQ8kuc9pgc9s8qqaq=dirpe0xb9q8qiLsFr0=vr0=vr0dc8meaabaqaciaacaGaaeqabaqabeGadaaakeaacqqGgbGrcqGHsislcqqGKbazcqqGPbqAcqqGZbWCcqqG0baDcqqGHbqycqqGUbGBcqqGJbWycqqGLbqziiaacqWF9aqpdaWcaaqaaiabbgdaXaqaaiabb6gaUbaadaaeqaqaaiabikdaYiabdAgaMnaaBaaaleaacqWGPbqAaeqaaOWaaqWaaeaacqWGybawdaWgaaWcbaGaemyAaKgabeaakiabgkHiTiabdMfaznaaBaaaleaacqWGPbqAaeqaaaGccaGLhWUaayjcSdaaleaacqWGPbqAaeqaniabggHiLdaaaa@4D9A@

where the weight

2fi=2×|0.5−∑NXiN|
 MathType@MTEF@5@5@+=feaafiart1ev1aaatCvAUfKttLearuWrP9MDH5MBPbIqV92AaeXatLxBI9gBaebbnrfifHhDYfgasaacH8akY=wiFfYdH8Gipec8Eeeu0xXdbba9frFj0=OqFfea0dXdd9vqai=hGuQ8kuc9pgc9s8qqaq=dirpe0xb9q8qiLsFr0=vr0=vr0dc8meaabaqaciaacaGaaeqabaqabeGadaaakeaacqaIYaGmcqWGMbGzdaWgaaWcbaGaemyAaKgabeaakiabg2da9iabikdaYiabgEna0oaaemaabaGaeGimaaJaeiOla4IaeGynauJaeyOeI0YaaSaaaeaadaaeqaqaaiabdIfaynaaBaaaleaacqWGPbqAaeqaaaqaaiabd6eaobqab0GaeyyeIuoaaOqaaiabd6eaobaaaiaawEa7caGLiWoaaaa@4268@

represents how far dimension *i *is from randomness in the information theoretic sense with respect to the distribution of the dimension over the entire N vectors. That is, *f*_*i *_empirically indicates how important feature *i *is in calculating the distance between two FEATURE vectors. The values of *f*_*i *_are calculated from the distribution of ones and zeros in feature *i *over the entire two million FEATURE vectors used.

### Characterization and validation of clusters

We created a histogram of cluster size for the resulting clusters and computed both the inter- and intraclass distances between vectors within each cluster to assess the degree of separation achieved in the clustering.

We annotated every residue in the data set that is part of a PROSITE pattern with that pattern's identifier. This results in an average of 6.2 ± 3.8 vectors per PROSITE hit. In order to biologically validate some of the clusters, we looked for PROSITE patterns for which at least 75% of the hits in our dataset are contained within a relatively small number of clusters and for which the pattern is the dominant annotation present in those clusters.

The clusters are available to the public for additional analysis and collaboration [27]

## Competing interests

The authors declare that they have no competing interests.

## Authors' contributions

SY, JCE, and EYC devised the algorithm, developed the code, performed the experiments, and drafted the manuscript. GDM and RBA provided guidance on the entire study and revised the manuscript.

## Figures and Tables

**Table 1:** Description of the 44 FEATURE properties

Dimension	Description	Dimension	Description
1	Aliphatic carbon	23	Residue name is ALA
2	Aromatic carbon	24	Residue name is ARG
3	Carbon with partial positive charge	25	Residue name is ASN
4	Aliphatic carbon next to a polar atom	26	Residue name is ASP
5	Amide carbon	27	Residue name is CYS
6	Carboxyl carbon	28	Residue name is GLN
7	Amide nitrogen	29	Residue name is GLU
8	Positively charged nitrogen	30	Residue name is GLY
9	Aromatic nitrogen	31	Residue name is HIS
10	Amide oxygen	32	Residue name is ILE
11	Carboxyl oxygen	33	Residue name is LEU
12	Hydroxyl oxygen	34	Residue name is LYS
13	Sulfur	35	Residue name is MET
14	Partial charge	36	Residue name is PHE
15	Van der Waals volume	37	Residue name is PRO
16	Charge	38	Residue name is SER
17	Negative charge	39	Residue name is THR
18	Positive charge	40	Residue name is TRP
19	Charge on histidines	41	Residue name is TYR
20	Hydrophobicity	42	Residue name is VAL
21	Solvent accessibility	43	Residue name is HOH
22	Number of atoms	44	Residue name is other
